# Liquid Crystalline Nanoparticles for Nasal Delivery of Rosuvastatin: Implications on Therapeutic Efficacy in Management of Epilepsy

**DOI:** 10.3390/ph13110356

**Published:** 2020-10-30

**Authors:** Mohammad Zubair Ahmed, Urooj A. Khan, Abdul Haye, Nidhi B. Agarwal, Nabil A. Alhakamy, Hani A. Alhadrami, Musarrat Husain Warsi, Gaurav K. Jain

**Affiliations:** 1 Nanoformulation Research Laboratory, Department of Pharmaceutics, School of Pharmaceutical Education and Research, Jamia Hamdard, New Delhi 110062, India; zubair_amgaz@hotmail.com (M.Z.A.); uakhan.sch@jamiahamdard.ac.in (U.A.K.); 2 Department of Pharmacology, School of Pharmaceutical Education and Research, Jamia Hamdard, New Delhi 110062, India; abdulhaye_sch@jamiahamdard.ac.in; 3 Centre for Translational and Clinical Research, School of Chemical & Life Sciences, Jamia Hamdard, New Delhi 110062, India; nidhi.bharal@gmail.com; 4 Department of Pharmaceutics, Faculty of Pharmacy, King Abdulaziz University, Jeddah 21589, Saudi Arabia; nalhakamy@kau.edu.sa; 5 Department of Medical Laboratory Technology, Faculty of Applied Medical Sciences, King Abdulaziz University, P.O. Box 80402, Jeddah 21589, Saudi Arabia; hanialhadrami@kau.edu.sa; 6 Special Infectious Agent Unit (Biosafety Level 3), King Fahd Medical Research Centre, P.O. Box 80402, Jeddah 21589, Saudi Arabia; 7 Department of Pharmaceutics and Industrial Pharmacy, College of Pharmacy, Taif University, Taif-Al-Haweiah 21974, Saudi Arabia; mvarsi@tu.edu.sa; 8 Department of Pharmaceutics, Delhi Pharmaceutical Science and Research University, Govt. of NCT of Delhi, Pushp Vihar, Sector III, New Delhi 110017, India

**Keywords:** rosuvastatin, epilepsy, liquid crystalline nanoparticles, seizures, status epilepticus, intranasal delivery

## Abstract

In the present study we investigated the protective role of intranasal rosuvastatin liquid crystalline nanoparticles (Ros-LCNPs) against pentylenetetrazole (PTZ) induced seizures, increasing current electroshock (ICES) induced seizures, and PTZ-induced status epilepticus. From the dose titration study, it was evident that intranasal rosuvastatin (ROS), at lower dose, was more effective than oral and intraperitoneal ROS. The Ros-LCNPs equivalent to 5 mg/kg ROS were developed by hydrotrope method using glyceryl monooleate (GMO) as lipid phase. The high resolution TEM revealed that the formed Ros-LCNPs were cubic shaped and multivesicular with mean size of 219.15 ± 8.14 nm. The Ros-LCNPs showed entrapment efficiency of 70.30 ± 1.84% and release was found to be biphasic following Korsmeyer–Peppas kinetics. Intranasal Ros-LCNPs (5 mg/kg) showed significant increase in latency to PTZ-induced seizures and ICES seizure threshold compared to control and intranasal ROS solution. Additionally, intranasal Ros-LCNPs provided effective protection against PTZ-induced status epilepticus. No impairment in cognitive functions was observed following intranasal Ros-LCNPs. The results suggested that Ros-LCNPs could be an effective and promising therapeutics for the epilepsy management.

## 1. Introduction

Epilepsy is one of the most severe neurological disease affecting nearly 80 million people worldwide [1]. Despite many therapeutic advancements in the recent years, the continued rise in epilepsy incidences has driven the need to develop novel therapeutic strategies with higher efficacy. The 3-hydroxy-3-methylglutaryl-CoA (HMG-CoA) reductase inhibitors (statins) are potent cholesterol-lowering drugs which could potentially be repurposed as an alternative for the management of epilepsy [2]. Statins have shown pleiotropic effects including anti-oxidant, immunomodulatory, anti-inflammatory, and anti-excitotoxic effects. Several in vitro and in vivo studies have demonstrated the neuroprotective effects of statins in Alzheimer’s disease, Parkinson’s disease, multiple sclerosis, cerebral ischemia, traumatic brain injury and in epilepsy. Statins anti-seizure activity has been previously investigated. A cohort study showed that mortality risk in patients with status epilepticus was reduced in patients receiving statins [3]. In another study, early long-term statin treatment has anti-epileptogenic effects in experiments performed on WAG/Rij rats (absence epilepsy animal genetic model) [4]. Most of the published reports demonstrated the anticonvulsant effects of statins, particularly atorvastatin, following intravenous or oral doses at high concentrations [5,6,7,8]. The present study evaluates the anti-epileptic effects of intranasal rosuvastatin (ROS). Compared to atorvastatin, ROS is more potent HMG-CoA reductase inhibitor and HMG-CoA reductase activity is reported to be correlated with anti-epileptic effects. HMG-CoA reductase action of statins has influence on nitric oxide metabolism, which is known to modulate synaptic transmissions [9]. Further, intranasal route offers myriads of advantages over oral and intravenous administration including possibility of self-administration, higher patient compliance, avoidance of gastrointestinal degradation and hepatic metabolism, avoidance of blood-brain barrier, and exploitation of nose to brain (olfactory) pathway to deliver the drug directly and rapidly to the brain [10,11].

Similar to other statins, ROS possesses poor pharmaceutical properties including low aqueous solubility (~17 µg/mL), poor oral bioavailability (~20%), high first pass metabolism and poor permeation via blood brain barrier [12]. This requisite a suitable formulation development even for intranasal delivery. Therefore, in the present study liquid crystalline nanoparticles (LCNPs) of ROS were developed. LCNPs are microstructural self-assembled liquid crystalline particles consisting of 3-dimensional structures with surfactant molecules honeycombed into bicontinuous domains of lipid and water. LCNPs show brilliant solubilizing properties, high drug loading capacity for a variety of sparingly water-soluble drugs, bioadhesion, controlled release owing to their small pore size and hence increased bioavailability [13,14,15]. Further, LCNPs could also cross mucosal barrier resulting in improved permeation. Beneficial effects of intranasal LCNPs has been demonstrated previously [16]. The present study explores the neuroprotective effect of intranasal Ros-LCNPs against seizures induced by pentylenetetrazole (PTZ), increasing current electroshock (ICES), and in status epilepticus [17]. Further, the effect of intranasal Ros-LCNPs on cognitive impairment was assessed to evaluate their preliminary safety profile.

## 2. Results

### 2.1. Intranasal Dose Titration

**Figure 1:**
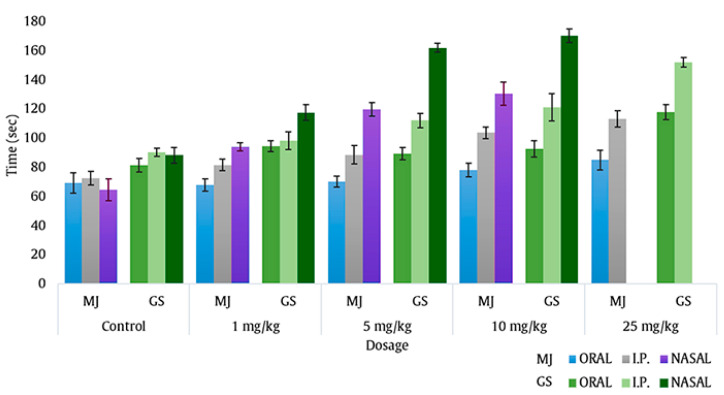
Effect of oral, i.p. and nasal route of administration of intranasal rosuvastatin (ROS) on mean latency to pentylenetetrazole (PTZ)-induced seizures; *p* > 0.5, nasal 5 mg/kg vs. nasal 10 mg/kg; *p* < 0.001, control nasal vs. nasal ROS 5 mg/kg; *p* < 0.001 control i.p. vs. i.p. ROS 25 mg/kg.

For intranasal dose selection, the latency time of 1, 5, 10 and 25 mg/kg dose of ROS administered via oral, intraperitoneal (i.p.) and intranasal route against pentylenetetrazole induced seizure were compared and summarized in Figure 1. The results showed that, irrespective of route of administration, the protective effect of ROS for both myoclonic jerks (MJ) and generalized seizures (GS) was dose dependent and an increase in latency time was observed with increase in ROS dose. The latency time, 94.2 ± 2.8 s for MJ and 116.5 ± 5.3 s for GS, showed by intranasal ROS at dose 1 mg/kg were not significantly different from those observed for 25 mg/kg oral ROS (85.0 ± 6.8 s for MJ and 118.4 ± 4.9 s for GS) and for 5 mg/kg i.p. ROS (88.5 ± 6.3 s for MJ and 112.0 ± 4.9 s for GS). The latency time observed for intranasal ROS at dose 5 mg/kg were more pronounced (119.8 ± 4.6 s for MJ and 161.9 ± 3.2 s for GS) and were comparable to those shown by 25 mg/kg i.p. ROS (113.1 ± 5.5 s for MJ and 151.8 ± 3.2 s for GS). Further, the latency time observed by 5 mg/kg intranasal ROS were not statistically different (*p* > 0.5) from those observed by 10 mg/kg intranasal ROS. From the data it was evident that latency to MJ and GS was higher by intranasal ROS at lower dose level compared to oral and i.p ROS. Thus, intranasal ROS at dose of 5 mg/kg was considered optimum and was selected for further formulation development.

### 2.2. Formulation Development of Ros-LCNPs

#### 2.2.1. Ethanol Concentration

Table 1 represents the effect of ethanol concentration on Ros-LCNPs. Ethanol concentration has negative effect on particle size and with increase in ethanol concentration from 0.5% (*w*/*v*) to 1.5% (*w*/*v*) the particle size of Ros-LCNPs decreases from 503.32 ± 35.15 nm to 186.31 ± 8.73 nm. The particles formed at 1% (*w*/*v*) ethanol concentration depicted lowest polydispersity index (PDI) and highest entrapment efficiency. With increase in ethanol concentration to 1.5% (*w*/*v*) both PDI and entrapment efficiency of LNCPs decreases and thus for Ros-LNCPs, 1% *(w*/*v*) of ethanol was considered as optimum.

#### 2.2.2. Lipid Concentration

The concentration of Glyceryl Monooleate (GMO), significantly affected particle size as well as entrapment efficiency and on increasing the quantity of GMO from 2.5% (*w*/*v*) to 5% (*w*/*v*), increase in particle size and entrapment was observed. However, the increase in size is comparative less as compared to increase in ROS entrapment. Further, at higher GMO concentration (7.5%, *w*/*v*) larger LCNPs were formed with low drug entrapment (Table 1). Thus, GMO at concentration of 5% (*w*/*v*) afforded small particles (219.15 ± 8.14 nm) with maximum drug entrapment (70.30 ± 1.84%) and was considered optimized.

#### 2.2.3. Stabilizer Concentration

Data in Table 1 showed that with increase in polaxamer concentration a significant decrease in particle size and PDI and a significant increase in entrapment efficiency was observed. LNCPs formed using 1 %, *w*/*v* of polaxamer depicted small particle size (219.15 ± 8.14 nm) and highest entrapment (70.30 ± 1.84 %). Increase in polaxamer beyond 1% resulted insignificant difference in LNCPs size however a decrease in entrapment was observed and therefore 1 %, *w*/*v* of polaxamer was considered optimized.

#### 2.2.4. Sonication Time

For optimization of sonication time, sonication amplitude was kept constant at 30%. Upon increase of sonication time up to 60 s, a significant reduction in LNCPs size and PDI and a significant improvement in drug entrapment was observed. However, further increasing sonication time to 90 s resulted in increase in particle size and a decrease in entrapment efficiency. Based on the data, sonication time of 60 s was considered as optimized.

**Figure 2:**
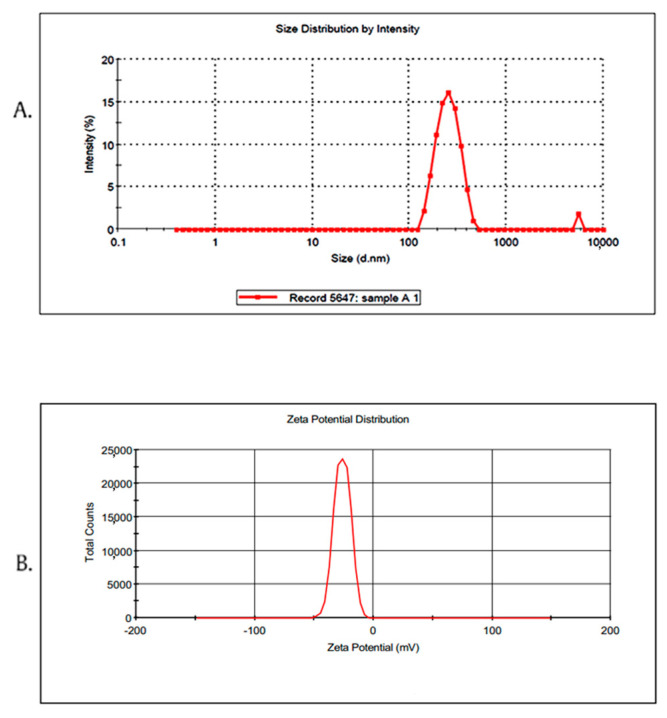
Optimized Ros-LCNPs (**A**) mean particle size, 219.10 nm; polydispersity index (PDI), 0.214; (**B**) zeta potential, −26.2 mv with 100% peak area.

The mean particle size of the optimized Ros-LCNPs was found to be 219.15 ± 8.14 nm (Figure 2A). Low polydispersity index (PDI) values of 0.24 ± 0.03 and the zeta potential values of −25.8 ± 0.67 mV, indicate formulation homogeneity and stability (Figure 2B), respectively. The entrapment efficiency of the optimized Ros-LCNPs was found to be 70.30 ± 1.84%, whereas drug loading was found to be 16.52 ± 2.12% *w*/*w*.

### 2.3. Characterization of Ros-LCNPs

#### 2.3.1. High-Resolution Transmission Electron Microscopy (HR-TEM)

**Figure 3:**
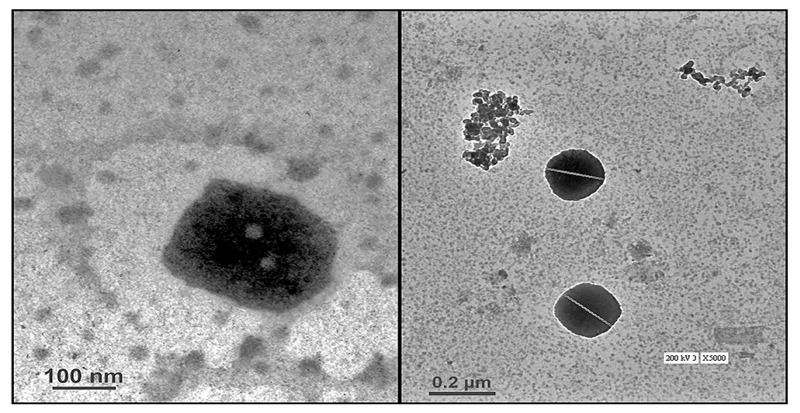
HR-TEM micrograph of Ros-LCNPs.

HR-TEM was conducted to study the morphology and size of the optimized Ros-LCNPs. The result revealed that Ros-LCNPs have discrete cubic symmetry shape (Figure 3) with mean particle size of 182.5 ± 10.26 nm. Further, the HR-TEM micrograph shows that the formed Ros-LCNPs are multivesicular.

#### 2.3.2. Drug Release and Kinetics

**Figure 4:**
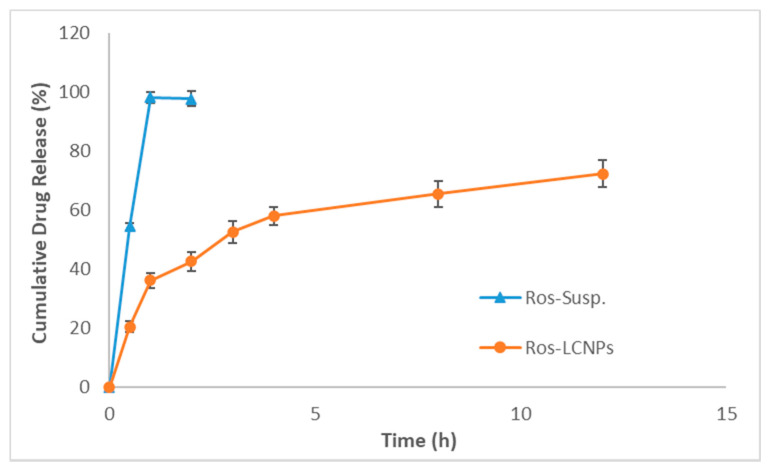
Release profile of Ros-LCNPs compared with ROS suspension (Ros-Susp).

The comparative release profile of the Ros-LCNPs and ROS suspension (Ros-Susp), with both the formulations having dose equivalent to 5 mg ROS, was shown in Figure 4. Ros-Susp showed immediate release profile with 98.2 ± 2.5% ROS release within 1 h. In contrast, Ros-LCNPs showed biphasic release profile with initial burst release of 36.2 ± 2.5% ROS in 1 h followed by a comparatively slower drug release thereafter releasing about 72.4 ± 4.2% ROS over 12 h time (Figure 4). The release kinetics showed that release of ROS from the Ros-LCNPs follows Korsmeyer–Peppas kinetics as suggested by higher R^2^ value of 0.9029. The value of release exponent ‘n’ was found to be 0.17, which indicates Fickian diffusion of ROS from LCNPs [18].

### 2.4. Effect of Intranasal Ros-LCNPs on PTZ-Induced Seizures

Table 2 shows the results of the PTZ-induced seizure studies. In the untreated group, the latency to myoclonic jerk and generalized seizure was found to be 71.6 ± 2.8 and 92.0 ± 3.6 s, respectively. The latency to myoclonic jerks (69.5 ± 4.7 s) and generalized seizure (95.2 ± 5.4 s) shown by vehicle treated group was not significantly different (*p* > 0.5) from the untreated group. Intranasal Ros-sol and Ros-LCNPs increased the latency to myoclonic jerk (123.2 ± 6.2 and 168.8 ± 7.5 s, respectively) and generalized seizure (164.4 ± 4.3 and 241.5 ± 5.4 s, respectively). The values were significantly higher for Ros-sol (*p* < 0.001) and Ros-LCNPs (*p* < 0.0001), compared with untreated group. Noteworthily, the increase in latency to myoclonic jerk and generalized seizure was significantly higher (*p* < 0.001) for intranasal Ros-LCNPs compared to Ros-sol.

### 2.5. Effect of Intranasal Ros-LCNPs on ICES-Induced Seizures

The current at which tonic hind limb extension (HLE) occurred was observed for all the groups and the values are shown in Table 2. In an untreated group and vehicle treated group, HLE occurred at 14.67 ± 0.67 and 14.18 ± 0.85 mA, respectively. The results showed that intranasal Ros-LCNPs increased the seizure threshold current to HLE and showed protection against ICES-seizures compared to untreated (*p* < 0.0001) and vehicle treated group (*p* < 0.0001). The intranasal Ros-sol also increased the threshold current to HLE, however the effect was significantly (*p* < 0.001) pronounced for intranasal Ros-LCNPs.

### 2.6. Effect of Intranasal Ros-LCNPs on Status Epilepticus

In PTZ-induced status epilepticus, latency to generalized seizures and the duration of clonic seizures post administration of intraperitoneal ROS solution was found to be 4.15 ± 0.72 and 28.24 ± 3.14 min, respectively (Table 3). The values were not considerable different from those observed for untreated group (3.85 ± 0.48 and 27.54 ± 1.68 min, respectively), however the decrease in mortality rate from 100% to 83% was observed. In contrast, intranasal Ros-LCNPs (5 mg/kg) significantly (*p* < 0.0001) increased the latency time (21.39 ± 2.08 min), decreased the duration of clonic seizures (18.24 ± 2.36 min) and displayed effective protection against status epilepticus. Results of Table 3 also showed that the mortality rate was decreased to 33.3% (2/6) in the group treated with intranasal Ros-LCNPs compared to 100.0 % (6/6) mortality in the untreated group.

### 2.7. Effect of Intranasal Ros-LCNPs on Cognition

Confusion and memory loss (cognitive impairment) are considered as one of the major side effects of anti-epileptic drugs. To evaluate the effect of intranasal Ros-LCNPs on cognitive impairment, forced swim test, elevated plus maze test and passive avoidance response test were performed. The results obtained are summarized in Table 4. The acquisition and retention latency time in passive avoidance response test and elevated plus maze test was not significantly different (*p* > 0.5) for intranasal Ros-LCNPs compared to control group. Similarly, immobility time in forced swim test was not significantly different (*p* > 0.5) for intranasal Ros-LCNPs compared to control group. The results demonstrated that intranasal Ros-LCNPs did not cause cognitive impairment in the treated mice over the test conduction period.

## 3. Discussion

Despite many therapeutic advancements in the recent years, the continued rise in epilepsy incidences has driven the need to develop novel therapeutic strategies, with higher efficacy to manage this disease. Statins are one class of drug that could potentially be repurposed as an alternative for the management of epilepsy, preferably when administered through intranasal route [5,6,7,8,17]. This study evaluates the beneficial and protective role of intranasal ROS formulated as LCNPs, against PTZ-induced seizures, ICES-induced seizures and PTZ-induced status epilepticus. Our preliminary dose titration studies demonstrated that intranasal ROS increased latency to myoclonic jerk and generalized seizure in PTZ-induced mice model and intranasal ROS was found to be more effective at lower doses (5 mg/kg) compared to oral and i.p ROS. To achieve patient compliant intranasal administration, Ros-LCNPs were developed.

For Ros-LCNPs development, hydrotrope based method was used. This method requires low energy and results in consistent and stable particles compared to conventional high shear and high-pressure dispersion methods [19,20]. The formulation was optimized varying concentration of ethanol, GMO, poloxamer and sonication time. Ethanol solubilized the lipid, avoided its aggregation with surfactant solution during premixing and facilitated homogenous nucleation. At low ethanol concentrations (<0.5%) no clear particulate structure was formed whereas high ethanol concentrations (>1%) resulted in increased size and longer development time. GMO, lipid phase, results in formation of vesicular structures upon precipitation and hydration. Low GMO concentration (<5%), although resulted in smaller sized vesicles but ROS entrapment efficiency was low. On other hand higher GMO concentration (>5%) resulted in larger vesicle size. Poloxamer helps in stabilizing LCNPs and also has impact on ROS entrapment [18,21]. On increasing the concentration, a significant improvement in quality attributes was observed. Sonication time also play critical role in size and entrapment efficiency of vesicles and lower sonication time (<60 s) results in larger vesicles whereas higher sonication time (>60 s) results in reduced drug entrapment. Vesicle size, PDI and drug entrapment are important quality attributes considering intranasal delivery. Particles with size close to 200 nm had shown to improve nose to brain delivery through receptor-mediated endocytosis [10,12]. Further, high drug entrapment ensures delivery of small volumes as a perquisite for intranasal delivery. The optimized formulation prepared using 1% ethanol, 5% GMO, 1% poloxamer and sonication for 60 s at 30% amplitude resulted in cubic shaped, multivesicular LNCPs with mean particle size in the range 210–230 nm. The same has been confirmed by dynamic light scattering measurements and HR-TEM micrographs. The developed Ros-LCNPs were homogenous and stable as evident from PDI and zeta potential values. The Ros-LCNPs showed biphasic sustained release following Korsmeyer–Peppas kinetics and Fickian diffusion from the multivesicular LCNPs [18].

Animal studies showed that intranasal Ros-LCNPs showed protective effect against PTZ induced seizures, ICES and PTZ induced status epilepticus. Pharmacodynamic studies revealed that ROS delayed the onset of seizures in both PTZ induced seizures and ICES. Intranasal ROS solution significantly increased the onset time to myoclonic jerk and generalized seizures when compared with the control groups. Similarly, HLE threshold current was also increased in comparison with control and provided substantial protection against ICES (Table 3). This activity may be due to nasal administration, which is believed to overcome highly restricting blood brain barrier and make the drug accessible for direct brain absorption via olfactory/trigeminal nerve pathways [10]. These findings were in accordance with studies previously reported by us using intranasal pitavastatin [10]. Interestingly, intranasal Ros-LCNPs showed better protection in both the seizures when compared with intranasal ROS solution (*p* < 0.001). Such behavior could be correlated with the increased permeation of drug from LCNPs, increased lipophilicity due to GMO and thus enhanced affinity for blood brain barrier permeation and high capability of nano-sized lipid vesicles to filter through nasal membrane. The present study did not determine the anticonvulsant properties of ROS on molecular level; however, it was inferred that like other statins, ROS can work against seizures by implementing multiple pleiotropic effects which are independent of cholesterol lowering properties. The effect of intranasal Ros-LCNPs in emergency circumstances-PTZ induced epileptic status, was investigated since it is well established that drugs rapidly reaches brain following intranasal administration [22,23]. It was found that intranasal Ros-LCNPs (5 mg/kg) significantly improved the latency time, decreased the clonic seizures duration, reduced the mortality rate and thus presented effective protection against status epilepticus when compared to control group and intravenous treated group. The use of intranasal ROS can be a viable option in status epilepticus type of situations which require emergency management.

The cognitive impairment effect of intranasal Ros-LCNPs using forced swim test, elevated plus maze test and passive avoidance response test was assessed. It was done to access the potential risk of memory deterioration after statin treatment as it has been supported by numerous experiments previously [24,25,26]. In case of cognition studies, insignificant differences were observed when compared to control, which confirmed that ROS did not induced behavioral changes emphasizing the preliminary safety of intranasal Ros-LCNPs treatment.

## 4. Materials and Methods

### 4.1. Materials

Rosuvastatin calcium was received as gift sample from Ranbaxy Laboratories Ltd. (Gurgaon, India). Glyceryl monooleate (GMO) was obtained from Gattefosse Ltd. (Mumbai, India). Phenytoin, PTZ, PEG 400 and poloxamer 407 were purchased from Sigma-Aldrich (Mumbai, India). HPLC grade methanol and acetone were purchased from Merck (Mumbai, India). ROS solution was prepared by dissolving measured quantity of ROS in aqueous solution of PEG 400 (5%, *v*/*v*). Pentylenetetrazole and phenytoin solutions were made in standard saline.

### 4.2. Animals

All the experiments were performed using healthy Swiss albino male mice weighing 25–40 g. Mice were held in a temperature-controlled environment (18–24 °C) with 50–60% humidity in natural day and night light cycle, with free access to food and water. All research procedures were appropriately authorized and agreed with the guidelines laid by Committee for Control and Supervision of Experiments on Animals (CPCSEA). Institutional Animal Ethics Committee, Jamia Hamdard, New Delhi approved the study (Approval No. 1113/2015) in line with the guidelines.

### 4.3. Intranasal Dose Titration

PTZ induced seizure in mice was selected as a model for intranasal ROS dose titration, as this model is well established. Intranasal doses at 1, 5, 10 and 25 mg/kg concentration were compared with same concentrations administered via oral and intraperitoneal (i.p.) routes. Animals were divided into 3 groups, having 30 animals each and were further sub-divided into 5 subgroups as per dosing frequency: control, 1, 5, 10 and 25 mg/kg, with six mice in each subgroup (*n* = 6). For oral and i.p. groups, normal saline served as control whereas aqueous PEG-400 solution served as control for intranasal group. Seizures were induced by i.p. administration of PTZ at dose of 60 mg/kg, dissolved in distilled water to form 10 mL/kg volume. Post PTZ administration, the mice were housed individually in plexi-glass cages (20 × 20 × 30 cm) and the latency to myoclonic jerks and generalized seizures were observed. The experiments were performed on the seventh day of dosing, 30 min after the administration of ROS.

### 4.4. Formulation Development of Ros-LCNPs

LCNPs were prepared by hydrotrope-based method as described previously [27,28]. Briefly, 1% aqueous poloxamer solution was prepared by soaking of poloxamer in water (surfactant solution). Ethanol (1%) was added to molten GMO at 60 °C (5% of dispersion volume) to constitute the hydrotropic phase. The hydrotropic phase was then dropwise added to the surfactant solution with constant stirring at 1000 rpm for 24 h with gradual cooling and evaporation of ethanol. The resultant dispersion was probe sonicated for 60 s at 30% amplitude to form LCNPs. Ros-LCNPs were prepared by dissolving the ROS in GMO prior to constitution of hydrotropic phase. The concentration of GMO (2.5–7.5%) with respect to dispersion volume, poloxamer (0.5–1.5% *w*/*v*), ethanol (0.5–1.5% *w*/*v*) and sonication time (30–90 s) were varied to study their effect on size, PDI and encapsulation efficiency of LCNPs. Non-critical parameters such as stirring speed (1000 rpm), stirring time (24 h) and dispersion volume (10 mL) along with drug concentration were kept constant throughout optimization.

### 4.5. Characterization of Ros-LCNPs

#### 4.5.1. Particle Size, Polydispersity and Zeta Potential

Particle size, PDI and zeta potential of Ros-LCNPs was determined by dynamic light scattering using Zetasizer (Nano-ZS, Malvern Instruments, UK). Ros-LCNPs were dispersed in water and light scattering was monitored at 25 °C using 90° scattering angle. For zeta potential, disposable capillary cell with a capacity of 1 mL was used.

#### 4.5.2. Entrapment Efficiency and Drug Loading

Encapsulation efficiency and drug loading was determined by lysing the known weight of dried Ros-LCNPs by adding them to a known volume of methanol and determining amount of drug entrapped by the UV spectrophotometer (UV-1601, Shimadzu, Japan) at a wavelength of 243 nm. DL and EE was calculated by the following Formulas (1) and (2) [29,30,31]:

(1) $$\mathrm{Encapsulation}\;\mathrm{efficiency}\;\left(\%\right)=\frac{\mathrm{Amount}\;\mathrm{of}\;\mathrm{ROS}\;\mathrm{entrapped}\;\mathrm{in}\;\mathrm{LCNPs}}{\mathrm{Total}\;\mathrm{ROS}\;\mathrm{added}}\times 100$$

(2) $$\mathrm{Drug}\;\mathrm{loading}\;\left(\%\right)=\frac{\mathrm{Amount}\;\mathrm{of}\;\mathrm{ROS}\;\mathrm{entrapped}\;\mathrm{in}\;\mathrm{LCNPs}}{\mathrm{Total}\;\mathrm{weight}\;\mathrm{of}\;\mathrm{LCNPs}}\times 100$$

#### 4.5.3. Microscopic Evaluation Using Transmission Electron Microscopy (TEM)

A Morgagni 268D transmission electron microscope (Fei Electron Optics, Eindhoven, Netherland) functioning at 70 kV was employed to observe surface morphology and size of Ros-LCNPs. The sample was made by putting a drop over the carbon foil-coated 400-mesh copper panel, which previously was diluted 50 times with double distilled water immediately followed by negative dying making use of 1 percent phosphotungstic acid. Combined with diffraction modes, the bright field imaging was used to investigate the shape and size of the Ros-LCNPs at increasing magnification [27].

#### 4.5.4. Drug Release and Release Kinetics

The drug release of the optimized Ros-LCNPs (ROS equivalent to 5 mg/mL) was studied by dialysis method [32,33] and comparisons were made with Ros-Susp (ROS equivalent to 5 mg/mL). The release study was conducted after incorporating 1.0 mL of formulations in the dialysis bag (10–12 KDa, Hi-Media, India) and suspending the dialysis bag in 900 mL of simulated nasal fluid (pH 6.4) as dissolution medium. The rotation speed was kept at 50 rpm and temperature of 37 ± 0.5 °C was maintained throughout experiment. Aliquots (2 mL) were withdrawn at predesignated time intervals and equal volumes of fresh medium were added to keep the volume constant. Spectrophotometric method was used to determine ROS concentrations at various time intervals using Shimadzu UV-1601 spectrophotometer (Shimadzu Co. Ltd., Kyoto, Japan) and samples were analyzed at 243 nm λmax. The dissolution studies were done in six-replicates.

### 4.6. In Vivo Studies

#### 4.6.1. Administration Protocol

Animals were divided into various groups to access the effects of intranasal ROS on seizure activity, cognitive function and status epilepticus. Animals were divided into 4 groups each for PTZ-induced and ICES preventive assessment test. In both the test, first group received Ros solution (5 mg/kg, Ros-sol), second group received Ros-LCNPs (equivalent to 5 mg/kg ROS), third group received intranasal PEG aqueous solution (control-treated) and fourth group did not receive any treatment (control-untreated). All the formulations were administered in 10 microliter volume intranasally with the help of micropipette. The experiments were conducted on the seventh day of dosing for acute effects of Ros-LCNPs on seizures, 30 min after formulation administration.

For status epilepticus, animals were divided into three groups (*n* = 6). First group received intranasal Ros-LCNPs (equivalent to 5 mg/kg ROS), second group received i.p ROS solution (25 mg/kg, control-treated) and third group did not received any treatment (control untreated). The single dose protocol was used and the experiment was performed 30 min after formulation administration.

For cognitive evaluation, animals were divided into 2 groups (*n* = 6). First group received intranasal Ros-LCNPs (equivalent to 5 mg/kg ROS) and second group was left untreated as control (control untreated). The study was conducted 30 min after administration of the formulation on the 7th day of the dosing [17].

#### 4.6.2. PTZ-Induced Seizures

For inducing seizures, 60 mg/kg PTZ solution was given i.p. (dissolved in 10 mL/kg water) [34]. This PTZ dose produced seizures (myoclonic jerks/generalized seizures) without any mortality in animals, the latency of which was immediately analyzed 30 min after injecting. PTZ administration was done 30 min after formulation administration. The latency duration in the absence of any seizures was recorded as 30 min (1800 s) [35,36].

#### 4.6.3. ICES Test

The ICES method was conducted to determine the convulsogenic effect of the drug. In each group, six mice were used. Electroshock was implemented using an electro-convulsometer via an ear electrode (forceps style). The electro-shock is composed of one 0.2 s pulse with 2 mA/2 s growing linear frequency. For each mouse the current was carefully examined and documented at which hind limb extension (HLE) took place. If no HLE is detected at 30 mA, the shock was stopped and the cut-off current measured was ascribed for evaluation [10,37,38].

#### 4.6.4. Status Epilepticus (SE) Studies

SE was triggered by injecting PTZ into the loose skin at 80 mg/kg dose subcutaneously behind the neck [39]. In order to eliminate PTZ produced terminal tonic HLE, 40 mg/kg phenytoin sodium dissolved in saline (alkalinized) was administered intraperitoneally two hours earlier to PTZ administration at a volume of 0.01 mL/g body weight of mice. A 60 min seizure-free state was taken as protection. Thirty minutes post formulations, SE was induced and observations were made subsequently [10,39,40].

#### 4.6.5. Cognitive Assessment

##### Forced Swim Test

Mice were tested 24 h before control treatment and 60 min post formulation treatment. In a plexiglass cylinder with diameter of 10 cm and height 25 cm having 9 cm of water, mice were lowered one by one and left to swim for a time period of 15 min [17,41]. They displayed a stance of inactivity after a short, vigorous exertion by floating motionlessly and making only those movements appropriate to hold the head above the water level. Each mouse was noted for 15 min for immobility having allowed one minute of acclimatization time. Thus, immobility time (i.e., total idle time in 5 min) was logged for each mouse.

##### Passive Avoidance Response

Negative reinforcement derived passive avoidance behavior was observed to analyze long-term memory. Apparatus for passive avoidance having a grid floor with electric supply to provide shock was used. In place of the hanging pole for a shock free zone as in Cook’s apparatus, on the center a wooden block was placed that functioned as shock-free zone. To measure the step down latency (SDL), 1 mA electric shock (20 V, 50 Hz) was applied for 1 s when the mice got off the wooden block and put all of its paws on the floor [42,43]. The time taken by the mouse to come down to grid floor from wooden platform with all its paws was termed as SDL. Mice with SDL within the range of 2–15 s in the first test were used for the second spell and the retention test. 90 min after initial test, the second session was conducted. During the second test if the mice stepped down before 60 s, electric shocks were again given for 15 s and if they did not step down for 60 s, they were manually taken out of the shock-free region. Memory persistence was similarly tested after 24 h, in absence of electrical shocks on the grid floor. This was termed as acquisition latency. The SDL was recorded placing each mouse on the platform again in absence of electric shock. 600 s was considered as upper cutoff time.

##### Elevated Maze Test

This test consists of a maze having two closed arms (50 × 10 × 40 cm) with closed top and two open arms (50 × 10 cm) without any roof at the top. This setup was elevated 50 cm from the floor on a central platform with arms extending from it. The animals were positioned separately at any of the open arms end and allowed to have access to the closed arms. This acted as a model for exteroceptive behavior in the assessment of mice learning and memory. In such models the stimulus occurs outside of the body. If, in the course of training, the mice did not reach the closed arm within 180 s, it was gently forced into the closed arm. If during the primary screening the mice did not reach the secured arm within 180 s, they were omitted from the test. Before keeping them back in their cage, mice were made to observe the maze for 20 s after having reached the closed arm in order to familiarize them with the setup. The cognition was then reviewed after 30 min the same day, and the mice were retested 24 h after the first day of practice for memory retention. The time took by the mice to migrate from the open arm to the closed arm of the maze was reported as transfer latency (TL). 180 s was taken as cut-off time and animals that didn’t enter into the closed arm during that time were attributed TL of 180 s. A longer latency period indicated poor memory retention in comparison to considerably shorter latency times [43,44].

### 4.7. Statistical Evaluation

All scores were reported in terms of mean ± standard error of the mean (SEM) for each group of 6 animals (*n* = 6). One-way analysis of variance (ANOVA) followed by Student’s *t*-test was used to analyze the data and *p*-value smaller than 0.05 was considered as significant.

## 5. Conclusions

The LCNPs formulation for ROS has been optimized and characterized for their intranasal administration. Compared to oral and i.p. routes, intranasal Ros-LCNPs showed improved and beneficial neuroprotection against acute seizures. In addition, intranasal Ros-LCNPs displayed effective protection in emergency condition of status epilepticus and did not produce any cognitive deterioration. Thus, ROS holds promise for repurposing in the treatment and management of epilepsy. Our observations will foster new research disclosing the molecular mechanisms to establish the safety and efficacy of intranasal Ros-LCNPs in humans.

## Figures and Tables

**Table 1:** Effect of ethanol concentration, lipid concentration, Poloxamer 407 concentration and sonication time on particle size, polydispersity index (PDI) and entrapment efficiency of rosuvastatin liquid crystalline nanoparticles (Ros-LCNPs).

Concentration	Particle Size (nm)	PDI	Entrapment Efficiency (%)
**Effect of ethanol concentration (%, *w*/*v*)**			
0.5	503.32 ± 35.15	0.32 ± 0.02	76.63 ± 1.07
1	219.15 ± 8.14	0.24 ± 0.03	70.30 ± 1.84
1.5	186.31 ± 8.73	0.41 ± 0.02	45.90 ± 3.99
**Effect of lipid concentration (%, *w*/*v*)**			
2.5	176.96 ± 9.97	0.38 ± 0.02	27.17 ± 3.34
5.0	219.15 ± 8.14	0.24 ± 0.03	70.30 ± 1.84
7.5	395.63 ± 17.43	0.27 ± 0.04	68.93 ± 3.40
**Effect of Poloxamer 407 (%, *w*/*v*)**			
0.2	435.27 ± 15.97	0.34 ± 0.03	61.58 ± 2.19
0.5	381.30 ± 19.31	0.29 ± 0.02	66.45 ± 3.39
1.0	219.15 ± 8.14	0.24 ± 0.03	70.30 ± 1.84
1.5	210.06 ± 12.91	0.25 ± 0.06	67.77 ± 3.24
**Effect of sonication time (s)**			
30	446.47 ± 21.26	0.38 ± 0.03	74.07 ± 2.36
45	330.98 ± 10.48	0.32 ± 0.025	72.36 ± 2.71
60	219.15 ± 8.14	0.24 ± 0.03	70.30 ± 1.84
90	424.38 ± 16.67	0.22 ± 0.02	37.97 ± 5.45

**Table 2:** Effect of Ros-LCNPs on PTZ-induced seizures and ICES; Data are expressed as mean ± SEM; *n* = 6 in each group, ANOVA followed by Tukey’s test.

Treatment	Mean Latency to PTZ-Induced Seizures		ICES
	Myoclonic Jerks (s)	Generalized Seizure (s)	Threshold (mA)
Untreated	71.6 ± 2.8	92.0 ± 3.6	14.67 ± 0.67
Vehicle treated	69.5 ± 4.7 ^a^	95.2 ± 5.4 ^a^	14.18 ± 0.85
Ros-sol	123.2 ± 6.2 ^b^	164.4 ± 4.3 ^b^	21.20 ± 0.93 ^b^
Ros-LCNPs ^c^	168.8 ± 7.5 ****	241.5 ± 5.4 ****	27.78 ± 0.86 ****

^a^*p* > 0.5 control vs. vehicle treated; ^b^ *p* < 0.001 control vs. intranasal Ros-PEG sol ^c^ *p* < 0.0001 control vs. intranasal Ros-LCNPs; **** *p* < 0.001 intranasal Ros-PEG sol vs. intranasal Ros-LCNPs.

**Table 3:** Effect of Ros-LCNPs on status epilepticus; data are expressed as mean ± SEM; *n* = 6 in each group, ANOVA followed by Tukey’s test.

Treatment	Time Taken by Mice to Develop Generalized Seizures (Latency in min)	Duration (min)	Average Mortality (%)
**Untreated**	3.85 ± 0.48	27.54 ± 1.68	100%
**I.P. Ros-sol**	4.15 ± 0.72	28.24 ± 3.14	83%
Ros-LCNPs *	21.39 ± 2.08	18.24 ± 2.36	33%

n.d. = not done; * *p* < 0.0001 control vs. Ros-LCNPs formulation 5 mg/kg intranasally.

**Table 4:** Effect of Ros-LCNPs on passive avoidance response test (step down latency model), forced swim test and elevated plus maze test (transfer latency model). Data are expressed as mean ± SEM; *n*= 6 in each group, ANOVA followed by Dunnett’s test.

Treatment	Dose	Passive Avoidance Response Test		Forced Swim Test	Elevated Plus Maze	
		Acquisition Latency (s)	Retention Latency (s)	Immobility (s)	Acquisition Latency (s)	Retention Latency (s)
**Untreated**	NA	513 ± 28.34	554.7 ± 18.19	97.00 ± 3.44	22.33 ± 0.84	8.833 ± 0.60
**Ros-LCNPs**	5 mg/kg	523.8 ± 13.07	549.2 ± 21.41	95.56 ± 5.70	21.67 ± 1.70	10.67 ± 0.74

*p* > 0.5 control vs. intranasal Ros-LCNPs 5 mg/kg.

## Abbreviations

AEDs	antiepileptic drugs
BCS	Biopharmaceutical Classification System
EE	entrapment efficiency
GMO	Glyceryl Mono Oleate
HR-TEM	High-resolution transmission electron microscopy
HLE	hind limb extension
ICES	increasing current electroshock
LCNPs	liquid crystalline nanoparticles
PTZ	Pentylenetetrazole
PDI	polydispersity index
ROS	Rosuvastatin
Ros-LCNPs	Rosuvastatin liquid crystalline nanoparticles
Ros-Susp	rosuvastatin suspension
SDL	step down latency
TL	transfer latency

## References

[1] Eadie M.J. (2012). Shortcomings in the current treatment of epilepsy. Expert Rev. Neurother..

[2] O’Regan C., Wu P., Arora P., Perri D., Mills E.J. (2008). Statin Therapy in Stroke Prevention: A Meta-analysis Involving 121,000 Patients. Am. J. Med..

[3] Sierra-Marcos A., Alvarez V., Faouzi M., Burnand B., Rossetti A.O. (2015). Statins are associated with decreased mortality risk after status epilepticus. Eur. J. Neurol..

[4] Citraro R., Chimirri S., Aiello R., Gallelli L., Trimboli F., Britti D., De Sarro G., Russo E. (2014). Protective effects of some statins on epileptogenesis and depressive-like behavior in WAG/Rij rats, a genetic animal model of absence epilepsy. Epilepsia.

[5] Moezi L., Shafaroodi H., Hassanipour M., Fakhrzad A., Hassanpour S., Dehpour A.R. (2012). Chronic administration of atorvastatin induced anti-convulsant effects in mice: The role of nitric oxide. Epilepsy Behav. E&B.

[6] Funck V.R., de Oliveira C.V., Pereira L.M., Rambo L.M., Ribeiro L.R., Royes L.F., Ferreira J., Guerra G.P., Furian A.F., Oliveira M.S. (2011). Differential effects of atorvastatin treatment and withdrawal on pentylenetetrazol-induced seizures. Epilepsia.

[7] Uzum G., Akgun-Dar K., Aksu U. (2010). The effects of atorvastatin on memory deficit and seizure susceptibility in pentylentetrazole-kindled rats. Epilepsy Behav. E&B.

[8] Lee J.K., Won J.S., Singh A.K., Singh I. (2008). Statin inhibits kainic acid-induced seizure and associated inflammation and hippocampal cell death. Neurosci. Lett..

[9] Seker F.B., Kilic U., Caglayan B., Ethemoglu M.S., Caglayan A.B., Ekimci N., Demirci S., Dogan A., Oztezcan S., Sahin F. (2015). HMG-CoA reductase inhibitor rosuvastatin improves abnormal brain electrical activity via mechanisms involving eNOS. Neuroscience.

[10] Ashhar M.U., Ahmad M.Z., Jain V., Agarwal N.B., Ahmad F.J., Jain G.K. (2017). Intranasal pitavastatin attenuates seizures in different experimental models of epilepsy in mice. Epilepsy Behav. E&B.

[11] Choonara Y.E., Kumar P., Modi G., Pillay V. (2016). Improving drug delivery technology for treating neurodegenerative diseases. Expert Opin. Drug Deliv..

[12] Clementino A., Batger M., Garrastazu G., Pozzoli M., Del Favero E., Rondelli V., Gutfilen B., Barboza T., Sukkar M.B., Souza S.A. (2016). The nasal delivery of nanoencapsulated statins—An approach for brain delivery. Int. J. Nanomed..

[13] Karami Z., Hamidi M. (2016). Cubosomes: Remarkable drug delivery potential. Drug Discov. Today.

[14] Pan X., Han K., Peng X., Yang Z., Qin L., Zhu C., Huang X., Shi X., Dian L., Lu M. (2013). Nanostructed cubosomes as advanced drug delivery system. Curr. Pharm. Des..

[15] Spicer P.T. (2005). Progress in liquid crystalline dispersions: Cubosomes. Curr. Opin. Colloid Interface Sci..

[16] Kim D.-H., Jahn A., Cho S.-J., Kim J.S., Ki M.-H., Kim D.-D. (2015). Lyotropic liquid crystal systems in drug delivery: A review. J. Pharm. Investig..

[17] Sehar N., Agarwal N.B., Vohora D., Raisuddin S. (2015). Atorvastatin prevents development of kindling by modulating hippocampal levels of dopamine, glutamate, and GABA in mice. Epilepsy Behav. E&B.

[18] Kumbhar D.D., Pokharkar V.B. (2013). Engineering of a nanostructured lipid carrier for the poorly water-soluble drug, bicalutamide: Physicochemical investigations. Colloids Surf. A Physicochem. Eng. Asp..

[19] Boyd B.J., Whittaker D.V., Khoo S.-M., Davey G.J.I. (2006). Hexosomes formed from glycerate surfactants—formulation as a colloidal carrier for irinotecan. Int. J. Pharm..

[20] Gabr M.M., Mortada S.M., Sallam M.A. (2017). Hexagonal liquid crystalline nanodispersions proven superiority for enhanced oral delivery of rosuvastatin: In vitro characterization and in vivo pharmacokinetic study. J. Pharm. Sci..

[21] Muheem A., Shakeel F., Warsi M.H., Jain G.K., Ahmad F.J. (2017). A Combinatorial Statistical Design Approach to Optimize the Nanostructured Cubosomal Carrier System for Oral Delivery of Ubidecarenone for Management of Doxorubicin-Induced Cardiotoxicity: In Vitro-In Vivo Investigations. J. Pharm. Sci..

[22] Pardeshi C.V., Belgamwar V.S. (2013). Direct nose to brain drug delivery via integrated nerve pathways bypassing the blood–brain barrier: An excellent platform for brain targeting. Expert. Opin. Drug Deliv..

[23] Serralheiro A., Alves G., Fortuna A., Falcão A. (2014). Intranasal administration of carbamazepine to mice: A direct delivery pathway for brain targeting. Eur. J. Pharm. Sci..

[24] Ramkumar S., Raghunath A., Raghunath S. (2016). Statin therapy: Review of safety and potential side effects. Acta Cardiol. Sin..

[25] Kitzmiller J.P., Mikulik E.B., Dauki A.M., Murkherjee C., Luzum J.A. (2016). Pharmacogenomics of statins: Understanding susceptibility to adverse effects. Pharmgen. Personal. Med..

[26] Maji D., Shaikh S., Solanki D., Gaurav K. (2013). Safety of statins. Indian J. Endocrinol. Metab..

[27] Swarnakar N.K., Jain V., Dubey V., Mishra D., Jain N.K. (2007). Enhanced oromucosal delivery of progesterone via hexosomes. Pharm. Res..

[28] Spicer P.T., Hayden K.L., Lynch M.L., Ofori-Boateng A., Burns J.L. (2001). Novel Process for Producing Cubic Liquid Crystalline Nanoparticles (Cubosomes). Langmuir.

[29] Avachat A.M., Parpani S.S. (2015). Formulation and development of bicontinuous nanostructured liquid crystalline particles of efavirenz. Colloids Surf. B Biointerfaces.

[30] Yang Z., Peng X., Tan Y., Chen M., Zhu X., Feng M., Xu Y., Wu C. (2011). Optimization of the preparation process for an oral phytantriol-based amphotericin B cubosomes. J. Nanomater..

[31] Rizwan S.B., Assmus D., Boehnke A., Hanley T., Boyd B.J., Rades T., Hook S. (2011). Preparation of phytantriol cubosomes by solvent precursor dilution for the delivery of protein vaccines. Eur. J. Pharm. Biopharm..

[32] Ahirrao M., Shrotriya S.J.D. (2017). In vitro and in vivo evaluation of cubosomal in situ nasal gel containing resveratrol for brain targeting. Drug Dev. Ind. Pharm..

[33] Qian S., Wong Y.C., Zuo Z.J.I. (2014). Development, characterization and application of in situ gel systems for intranasal delivery of tacrine. Int. J. Pharm..

[34] Oliveira M.S., Furian A.F., Royes L.F., Fighera M.R., Fiorenza N.G., Castelli M., Machado P., Bohrer D., Veiga M., Ferreira J. (2008). Cyclooxygenase-2/PGE2 pathway facilitates pentylenetetrazol-induced seizures. Epilepsy Res..

[35] Vohora D., Pal S., Pillai K. (2000). Thioperamide, a selective histamine H3 receptor antagonist, protects against PTZ-induced seizures in mice. Life Sci..

[36] Löscher W., Schmidt D. (1988). Which animal models should be used in the search for new antiepileptic drugs? A proposal based on experimental and clinical considerations. Epilepsy Res..

[37] Marwah R., Pillai K., Pal S.N. (1998). Effect of Fluoxetine Alone and in Combination with Anticonvulsants on the Increasing-Current Electroshock Seizure Test.

[38] Kitano Y., Usui C., Takasuna K., Hirohashi M., Nomura M. (1996). Increasing-current electroshock seizure test: A new method for assessment of anti-and pro-convulsant activities of drugs in mice. J. Pharmacol. Toxicol. Methods.

[39] Raines A., Henderson T.R., Swinyard E.A., Dretchen K.L. (1990). Comparison of midazolam and diazepam by the intramuscular route for the control of seizures in a mouse model of status epilepticus. Epilepsia.

[40] Agarwal N.B., Jain S., Nagpal D., Agarwal N.K., Mediratta P.K., Sharma K.K. (2013). Liposomal formulation of curcumin attenuates seizures in different experimental models of epilepsy in mice. Fundam. Clin. Pharmacol..

[41] Vogel H.G., Vogel W.H. (2013). Drug Discovery and Evaluation: Pharmacological Assays.

[42] Shafique A., Pillai K., Hasan A.S.A., Najmi A. (2013). Modulation of conditioned avoidance response and quetiapine-induced metabolic syndrome by rosuvastatin and CDP-choline in rats. J. Pharmacol. Pharmacother..

[43] Agarwal N.B., Agarwal N.K., Mediratta P.K., Sharma K.K. (2011). Effect of lamotrigine, oxcarbazepine and topiramate on cognitive functions and oxidative stress in PTZ-kindled mice. Seizure.

[44] Sharma A.C., Kulkarni S.K. (1992). Evaluation of learning and memory mechanisms employing elevated plus-maze in rats and mice. Prog. Neuro-Psychopharmacol. Biol. Psychiatry.

